# Child Well-Being in Times of Confinement: The Impact of Dialogic Literary Gatherings Transferred to Homes

**DOI:** 10.3389/fpsyg.2020.567449

**Published:** 2020-10-30

**Authors:** Laura Ruiz-Eugenio, Esther Roca-Campos, Susana León-Jiménez, Mimar Ramis-Salas

**Affiliations:** ^1^Department of Theory and History of Education, University of Barcelona, Barcelona, Spain; ^2^Department of Comparative Education and Education History, University of Valencia, Valencia, Spain; ^3^Department of Sociology, Faculty of Economics and Business, University of Barcelona, Barcelona, Spain

**Keywords:** coronavirus, successful educational actions, childhood, well-being, quality relationships

## Abstract

The COVID-19 pandemic has created an unexpected situation that has forced people to find educational alternatives to support learning and ensure child well-being. The need for practices that “open doors” at home as a way to promote a quality education and to foster an environment of supportive relationships and a sense of community, has led to the in-depth analysis of successful educational actions, particularly the Dialogic Literary Gatherings (DLGs). The aim of this article is to show how the transference of DLGs to the home environment has had an impact on child subjective well-being in times of confinement, promoting a safe and supportive environment for learning, interacting, coexisting and on emotional development at different educational stages, especially for the most vulnerable children. Data collection consisted of a focus group of 10 teachers, 6 semi-structured interviews addressed to families and 6 life stories of students, from 4 primary education centers, 1 high school, and 1 Special Education School. Communicative methodology structured the two-level data analysis, for studying both the elements provided by online DLGs that favor and achieve child well-being, and the elements that may hinder those achievements. The results confirm that DLGs have had a notorious impact on children’s and their families’ well-being. Considering the findings in the development of educational public policies and the possibility of extending “open doors actions” as an option for future learning environments beyond the confinement situation is contemplated. Future research on how these spaces can have an impact on child well-being in upcoming contexts of the new normal in the education domain will be of interest.

## Introduction

The COVID-19 pandemic crisis has created an unexpected situation that has forced people to interrupt everyday activity to avoid the disease transmission, with schools closing as an emergency action (Enserink and Kupferschmidt, 2020; Wang et al., 2020). The way in which the outbreak is tackled may imply critical long-term effects (Gates, 2020), what is required is the implementation of successful procedures to reduce to the extent of any possible undesirable effects, especially for the most vulnerable children (Brooks et al., 2020). In this context, the need to give continuity to teaching and learning, despite school suspension, through online education (Zhang et al., 2020), and to rapidly find educational alternatives for supporting this learning and ensuring child well-being has become an urgent necessity. Children are more vulnerable than adults in the face of traumatic situations and their impact on their daily routines (Bartlett et al., 2020). The quickly evolving new situation exposes children to conversations, constant media information, anxiety-inducing environments and changes and continuous adjustments to their routines as a result of the outbreak (Dalton et al., 2020). The global situation in general, and the prolonged home confinement, the social distancing and the school closures in particular, are having particular negative consequences on children’s mental and physical health, interfering with their sense of security, structure or predictability (Bartlett et al., 2020; Wang et al., 2020), both in the short and long term, which cannot be neglected (Dalton et al., 2020). Regarding the mental effects, a psychological impact on anxiety, fear, boredom or frustration, *inter alia* (Brooks et al., 2020) has been reported, as well as trauma both for children and their families (Sprang and Silman, 2013). This situation can become a vicious circle, as the subsequent social-emotional and behavioral disorders may also contribute to more adverse results on health such as cardiovascular diseases, excessive weight gain, poor quality of life (Perrin et al., 2016), and a risk for future mental illnesses and cognitive development (Decosimo et al., 2019). These health consequences become more significant and persistent when we talk about vulnerable children with previous trauma or preceding physical, mental, or developmental disorders, as well as those with problems within the family (Bartlett et al., 2020). The lack of interactions with friends and outdoor activities (Wang et al., 2020), the absence of personal space at home, or the family’s financial problems, are among other factors affecting child well-being in confinement, which have a challenging impact on youth and child well-being (Brooks et al., 2020). But despite the contingencies caused by this outbreak, especially on education around the world, this interruption is also providing new opportunities to find and discover transformative and stimulating practices, where the whole community—families and teachers—with the support of institutions and administrations, meet and reinvent education for the sake of the continuation of learning (Dryden-Peterson, 2020).

Organizations like the World Health Organization–UNICEF–Lancet Commission agree that children must be placed at the center of the Sustainable Development Goals (Clark et al., 2020), an idea that becomes imperative in the new situation. The Children’s Bureau of the U.S. Department of Health and Human Services, through the Child Welfare Information Gateway, puts the focus on family support and engagement and community-based practices to ensure children’s safety and well-being (Child Welfare Information Gateway, 2020). Those activities and the resources developed for promoting healthy behaviors in children should take under consideration some preconditions, such as protecting children in the face of online exposure, considering minimal equipment and small spaces, and guaranteeing opportunities for family-child interactions (Guan et al., 2020).

Child well-being is imperative in the current situation, in the wider present, and in the future as it will influence and predict forthcoming outcomes as adults (Lansford et al., 2019). The literature on child well-being is large and keeps on expanding, and therefore several and different definitions and indicators can be found, what makes the research review and the choice for a definition more complex (Pollard and Lee, 2003; Statham and Chase, 2010). From science, research projects, governments, institutions, organizations such as OECD or UNICEF and committees of the European Union or the United States, *inter alia*, there have been different attempts to create a system of indicators for measuring child well-being. Nevertheless, more work and attention on this is needed (Moore, 2020). In order to understand the concept, a multidimensional notion of child well-being definition seems the most suitable to approach a holistic reality incorporating physical, mental and social aspects of the person (Statham and Chase, 2010), influenced by the interactions with family, peers, the community, but also by policies and programs (Lansford et al., 2019). Different studies provide their classifications of child well-being domains, but many of them coincide to focus on cognitive, physical, psychological, educational, social or behavioral domains to define and measure the feeling of wellness in children (Pollard and Lee, 2003; Bradshaw and Richardson, 2009; Lansford et al., 2019). The latest tendencies in measuring child well-being have gradually evolved into engaging children in defining their perception and interpretation of well-being, what emphasizes the importance of subjective well-being in studies (Statham and Chase, 2010) and the consideration of a communicative perspective of research through the inclusion of the participants’ voices (Flecha, 2000; Puigvert et al., 2012). This study will focus on the latter, paying special attention to the subjective well-being dimension as the sense of happiness or life satisfaction (Dinisman and Ben-Arieh, 2016), where the supportive interpersonal relationships constitute one of the main predictors (Diener et al., 2018). It will also measure it from a communicative perspective, considering the participants’ view through the voices of families and teachers, but mainly focusing on students.

The role of schools in child well-being is decisive for its educational and psychological aid (Wang et al., 2020). Broader environments or connection to community settings have a major role in overcoming childhood adversities and in resilience (Bartlett et al., 2020). Health depends on the contexts that nurture both physical and mental wellbeing, and healthy environments go beyond the home, including the community or the school, implying stable relationships and interactions or the development of learnings and skills through education, among others (Center on the Developing Child, and Harvard University, 2014).

Thus, the need for actions that “open schools’ doors” at home to promote a quality education and to foster an environment of supportive relationships and a sense of community, reinforcing child development and mental health (Roca, 2020) and improving the social, emotional and academic dimensions has led to the in depth analysis of the implementation of successful educational actions (Flecha, 2015) in confinement through virtual means. Thus, some schools have started to implement *Open Doors Actions*: actions that emerge from new developments and from the transference of identified evidence-based strategies, aimed at educational communities, families, teachers and mainly students, that traditionally have created a safe and friendly environment for improving learning and coexistence in schools, and which are now brought through a virtual medium to home spaces. These actions for training, sharing and exchanging are forming a response to the challenge of maintaining caring, rich, and supportive interactions among peers and their educational communities. Evidence reported from eight schools has shown a positive impact on the emotional and cognitive development of children in confinement (Roca et al., 2020).

Among these evidence-based actions, one of the most extended practices are the Dialogic Literary Gatherings (DLGs), globally implemented in more than 3000 schools and centers around the world (Lopez de Aguileta, 2019). DLGs are dialogic spaces for educational purposes where participants—children and/or adults—involving egalitarian dialogs around classical readings of the universal literature, and where all of them have the same opportunities to speak without hierarchies, and creating in interaction new meanings about the text discussed (Flecha, 2000; García-Carrión et al., 2020). In them, participants delve into universal values, issues and feelings of concern that humankind has experienced since ancient times through the discussion of complex and rich works, finding meanings and reflecting on them upon the participants’ own experiences and building new interpretations all together. The functioning is as follows: all students read alone, at home or at school, a previously decided piece of text from the book being read, which can be a faithful and high-quality adapted version of the original classic work. They choose a paragraph and note down the reasons that led them to select it. Along the DLG session, the students who wish to share their chosen paragraph as well as their reasons, and all the students start a debate or discussion on the raised idea, with the aim being to reflect on and to share and build meanings according to the participants’ experiences (Flecha, 2009; Lopez de Aguileta, 2019). The egalitarian dialogue (Serrano et al., 2010); the diversity of voices to enrich interpretations and debate (Flecha, 2009); or the fact of grounding the contributions on arguments instead on of power claims (Oliver and Gatt, 2010) are crucial premises according to the dialogic learning principles (Aubert et al., 2008) executed when implementing DLGs.

Benefits of the DLGs have been shown in a wide range of contexts (Soler, 2015), and their impact can be found in different areas. In the academic field, the children involved have shown a boost in the school-relevant language, literacy skills (Lopez de Aguileta, 2019), reading skills, vocabulary acquisition, communicative skills (de Botton et al., 2014) and an increase of students talk ratio and quality participation through reasoning and argumentation (García-Carrión et al., 2020). Additionally, DLGs have nurtured transformations from a personal to a social and contextual level (Serrano et al., 2010), improving students’ and their families’ confidence and motivation for learning, as well as fostering community-school links, and transforming child-parents’ interactions at home, with the discussion on classical works becoming part of their routine (de Botton et al., 2014). Evidence is provided about their effectiveness at all stages of life and in every context where they are implemented in all their diversity: from rural communities, to extremely disadvantaged backgrounds, high-complexity schools, special education centers, children’s residential care institutions, adults educational centers or prisons (Pulido-Rodríguez et al., 2015; Alvarez et al., 2016; García-Yeste et al., 2017; Garcia et al., 2018; Rodrigues and Marini, 2018; Duque et al., 2020), to mention a few examples. The implementation of DLGs in said settings has been conducted on-site until confinement, but since the pandemic situation, the schools participating in the study are developing these meetings online, through a video conference platform, providing an opportunity for social contact during lockdown. The moderator continues to play a very important role both for maintaining a warm environment that encourages participation and for promoting dialogic principles. This study explores DLGs as an evidence-based action replicable in any educational on-site environments, which has been incorporated to European recommendations and public policies (Gómez et al., 2010; European Commission, 2011) and is now being transferred to online spaces. In this new situation, and considering all its benefits, this study seeks to explore two core issues: the extent to which the transference of DLGs to homes through online means can have a positive impact on child well-being in these times of confinement on the one hand, and how this learning action can promote a safe and supportive environment for learning, and how it can interact and coexist in different education stages, especially for the most vulnerable children. This is necessary not only to respond to the challenges of the current pandemic, but also for meeting future demands regarding the protection of child well-being and health (Shonkoff and Williams, 2020). In the framework of this study, the analysis has focused on the measurable areas of child well-being, focusing on prosocial behavior; positive relationships with family, peers and other adults as teachers; and academic performance, covering the behavioral, psychological, social and cognitive domains (Lansford et al., 2019).

## Materials and Methods

Little agreement is found in the literature when the best way to measure child well-being is discussed as diverse approaches are developed: from objective measures with assessments, records, tests, rates or statistics, to subjective measurements through interviews or scales, finding more subjective measures on literature than objective ones (Pollard and Lee, 2003). To this end, a communicative approach is used, through the joint construction of knowledge between researchers and research participants, what enables a better understanding of the improvements generated in the well-being of children due to the implementation of the DLGs in confinement times (Puigvert et al., 2012). The six DLGs, object of analysis, were already implemented in the participating schools before confinement and have been developed online through a video conference platform during confinement. Communicative methodology recovers the voices and views of the different educational agents- families, teachers and students themselves, for generating profound analysis with the aim of building useful knowledge for affording child well-being at present, as well as in future crises (Gómez et al., 2019).

### Research Site

The schools participating in the study are part of a sub-net of SaLeaCom school (Rodrigues and Marini, 2018), in the neighboring territories of the Valencia and Murcia regions in Spain. These schools are part of the *Open Doors Schools* (Roca et al., 2020) project that started on March 18, 2020, to foster learning and supportive relationships, as well as a safe environment for childhood. This research has been conducted with schools that had already implemented traditional DLGs in person, although this is not a requirement for implementing online DLGs, and neither is the prerequisite of having had families participating before in the face-to-face format. Among all the schools, those that had already been implementing the online DLGs for at least 3 weeks were chosen. All the schools participating in this study ensured access to technology for all their students before starting the implementation of DLGs.

All the participating schools are public ones. The participating schools are diverse and heterogeneous: there are 4 primary education schools (PS), a special education center (ES) and a high school (SS). See Table 1 for more detailed data on the characteristics of the participating schools.

**TABLE 1:** Participating schools’ data.

**School**	**Size (students)**	**Teaching staff (number)**	**+30% risk**	**Years DLG**	**Weeks ODA DLG**	**Times a week**
PS1	260	24		7	9	1
PS2	410	35		5	8	1
PS3	462	33		7	7	1
PS4	540	40	X	8	5	1
SS1	800	80	X	3	11	1
ES1	197	54	X	5	5	1

### Participants

With the aim of exploring the transference of DLGs to homes, the data reported in this study includes two communicative focus groups with teachers of the six schools, six semi-structured interviews with relatives and six communicative life stories with students, from the six schools, counting on: (a) one or two teachers per school in the focus group, a total of ten teachers; (b) one relative (mother or father) per school, a total of six, and (c) one child per school, a total of six, too. Three of the schools receive more than 30% of students in situation of vulnerability, including students with special needs and others at risk of social exclusion. Table 2 shows the details regarding the participants’ profiles and the data collection technique (fully explained in subsection “Data Collection and Techniques”) employed to gather the information (see Table 2).

**TABLE 2:** Participants’ profiles in each data collection technique.

**Profile**	**Age**	**School**	**School level**	**Time in the school (years)**	**Time participating in traditional DLGs in this school (years)**	**CFG**	**Int**	**LS**
PP1	41–45	PS1	2° PS	11	8	X		
TP2	41–45	PS2	6° PS	5	5	X		
TP2’	31–35	PS2	2° PS	2	2	X		
TP3	41–45	PS3	5° PS	2	2	X		
TP3’	51–55	PS3	4° PS	1	1	X		
TP4	41–45	PS4	3° PS	2	2	X		
TS1	46–50	SS1	1° baccalaureate	9	3	X		
CS1	46–50	SS1	1° baccalaureate	11	3	X		
TE1	31–35	ES1	Primary	8	5	X		
TE1’	31–35	ES1	Primary	5	5	X		
FP1	46–50	PS1	2° PS	7	7		X	
FP2	51–55	PS2	6° PS	10	2		X	
FP3	41–45	PS3	5° PS	7	1		X	
FP3’	41–45	PS3	5° PS	7	1		X	
FP4	51–55	PS4	3° PS	15	6		X	
FS1	41–45	SS1	1° baccalaureate	5	1		X	
FE1	41–45	ES1	Primary	5	5		X	
SP1	6–10	PS1	2° PS	5	5			X
SP2	11–15	PS2	6° PS	4	2			X
SP3	6–10	PS3	5° PS	8	6			X
SP4	6–10	PS4	3° PS	6	3			X
SS1	16–18	SS1	1° baccalaureate	6	1			X

### Ethics

All participants (teachers, families, and students) agreed to provide researchers with relevant data for the purpose of the study. Prior to data collection, they were informed of the nature of the investigation and informed written consent was obtained. In the case of minors, the informed consent of their parents or guardians was collected. All participants were informed that their participation was anonymous and voluntary, and that the data would be treated confidentially and used only for research purposes. The study respects the ethical guidelines of the European Commission (European Commission, 2013) and was approved by the Ethics Board of the Community of Researchers in Excellence for All (CREA)^1^.

In order to ensure anonymity, a code was assigned to each participant and school. For participants, the first letter corresponds to their educational profile: T (teacher), P (school principal) and C (school counselor), and the second letter corresponds to the stage or kind of school where they are enrolled: P (Primary school), S (Secondary school), or E (Special Education center). Finally, a correlative number is indicated. For schools, the coding is similar: PS (Primary School), SS (Secondary School) and ES (Special Education School), adding a correlative number for schools with the same profile.

### Data Collection and Techniques

Due to the confinement situation, the fieldwork was carried out in an online format between May 2 and May 24, 2020, after more than 3 weeks of implementing virtual DLGs usually for 1 h a week. The data collection was carried out through virtual means. A script was planned for the evidence collection, including questions about different blocks of contents: (a) exploration about the concerns and observations about the implementation of DLGs at schools and now at home, (b) the influence of the implementation of DLGs on well-being, and (c) new opportunities emanated from the transference of DLGs to virtual spaces. The focus group, as well as the interviews and the life stories were audio-recorded and transcribed.

#### Communicative Focus Groups (CFGs)

Two CFG sessions were held. The objective of CFGs was to develop a shared analysis of the situation under study. The first CFG was carried out with one representative per school, in which the objectives of the study were validated, the dimensions of the impact on child well-being were discussed, and consensus was established around the collection of information to analyze the impact. The second CFG was carried out with 10 teachers from the 6 schools participating in the study. It analyzed how DLGs were being recreated in each of the schools and in the diversity of educational stages. Later, an in-depth analysis was done exploring the aspects of child well-being that were being more susceptible to improvement thanks to the DLGs. The CFG is a moment of analysis that enables the dialogic construction of scientific knowledge by creating bridges between scientific evidence, the object of research, and educational practices (Aubert et al., 2011). In these spaces, not only are the results are identified, but the participating people discover improvements to introduce in their DLGs through dialogue with other teachers.

#### Interviews With a Communicative Approach (Int)

Six semi-structured interviews with a communicative approach were carried out with six mothers, one from each participating school. The objective of the interviews was to analyze how families appreciated the performance of DLGs in confinement, their impact on the well-being of their daughters and sons, and the extent to which they were a space of prevention and care for boys and girls. Families were able to relate details about how their children prepared for the DLGs, how they saw them before and after participating in them and the extent to which DLGs became part of their children’s lives, and also of other family members’ lives.

#### Life History (LH)

Six short life stories were carried out with six students, one for each participating school, five for the primary education stage, one for special education and one for the secondary education stage. The objective of carrying out life histories over a short period of time is to dialogically reconstruct the reality lived by the student, giving voice to their thoughts, feelings and analysis. A cooperative process of understanding their experiences in DLGs was aimed at caring for and improving their well-being and that of their peers.

### Data Analysis

The six DLGs under study were analyzed together to understand how they contributed to the improvement of children’s subjective well-being in the diversity of educational stages, contexts and characteristics of the students. Taking into account the challenges that confinement has posed for children and their families and the possible consequences for their later development (Decosimo et al., 2019; Dryden-Peterson, 2020; Wang et al., 2020), this study addresses the impact of DLGs, taking as a reference, indicators related to child well-being from a subjective perspective, such as (1) the educational dimension (the developments and improvements achieved in instrumental learning and cognitive development), (2) the social dimension (considering the relationships and interactions of quality, and the improvements achieved in social cohesion), and (3) the emotional dimension (feelings and affective development), as well as the (4) impact related to home and families, and (5) the barriers that have been identified to hinder those achievements and future perspectives.

## Results

The analysis shed light on indicators of child well-being where it is shown, from a subjective dimension counting on participants from the communities, how the DLGs online have impacted the children’s feelings of wellness and even their feelings toward home and families in confinement times. This study also takes into account the barriers and challenges of the new implementation of this action, so besides proposing all the transformative dimensions, an account on the barriers is also reflected. Thus, the “Results” section is divided on the transformative conditions according to child well-being dimensions, (a) educational; (b) social; (c) emotional factors, or (d) the impact on families as well as (c) the challenges emerged in this novel situation and future prospects, according to participants’ experience.

### Learning, Cognition and Performance: The Educational Indicator

The schools participating in this study implement DLGs once a week for 1 or 2 h during the pandemic. More and more students have joined the DLGs since the online transference and, despite the short time of application, participants agree on the positive potential that it is having on different dimensions of child-wellbeing, but also in all the educational stages and with the diversity of children, mainly for the most vulnerable ones, those with special educational needs.

Improvements on the educational field are very noticeable according to the participants. For teachers, advances on different dimensions of learning are shown more sharply along confinement activity. Advances on reading skills, as this teacher comments “I have seen very clearly a progress in the children’s reading performance. Since the confinement has started, they have improved a lot” (TP3); or in the linguistic production, very noticeable with the most vulnerable children, those from the Special education center, who were not able to build sentences further than a subject plus a verb, and now one of the school teachers praises “[In confinement] they have been incorporating the linguistic model into their language, creating longer structures.”

Enhancements at a learning level have also been shown in relation to the instrumental knowledge. The emanated relations that students establish between the read texts and other school subjects suppose a creation of meaning within learning contents. Teachers, families and students agree on the fact that the readings and debates are very helpful for instrumental learning, mainly in the contextualization of learning, vocabulary acquisition, development of thought and oral expression, without being hindered by the online medium.

Now, there is a creation of meaning, because you can send them [as task] the “length measurements” and they do it, but for example when in Tom Sawyer the yards come out, and they look for it [the meaning] and they know that they are 91 and a bit more centimeters… or the word “stunned,” or “dread.” It happens that the instrumental learning that you are providing them with online now [with the DLGs] acquires a meaning, that instrumental learning, with the DLGs, has more meaning (TP4).

As we are little children, there are difficult words in the books and there we can learn as we comment on them (…). We cannot go to school, we cannot see as much things as when we attend school and the DLGs help us to know more things (SP1). Although we do the DLGs at home, we learn the same (SP4).

The main thing is that it [DLGs] helps them with the vocabulary and to express what they think. Nowadays with so much technology this is has been a little forgotten, the oral aspect, the ideas. everything is writing in WhatsApp and I miss this (DLGs) in my older children. who have not had them (FP4).

Dialogic Literary Gatherings have also shown improvements regarding children’s habits, both in maintaining daily routines and even in boosting the reading habit itself. One student expresses how helpful they have been for her, not only for learning, but also for continuing the learning and reading routine in confinement time:

It is like in the school, to keep on the same habit, but through videocall (…) They [DLGs] helped me to read more (…) It is a funny way to express yourself while you learn new things, you read… It would be amazing that children who don’t do DLGs started to! (SP3).

The educational performance has seen benefits through a notorious increase in the students’ participation. All participating teachers agree on this improvement, reporting that changes in this direction are regarded as beneficial, especially for those who had more difficulties with engaging in debates:

Changes have been noted especially at the level of participation, children who did not participate in person, are more participatory in the online [format]…. What seems nice is that it is counteracted in another way, the children who found more difficult for participate [in the on-site DLGs] are having a very important benefit now (TP2).

### Interactions and Relations: The Social Dimension of Well-Being

According to the social dimension benefited by the DLGs, the fact of meeting online with the DLGs has helped students to better understand the new situation. One of the teachers who forms the special education center reports how for their students the new pandemic, and the subsequent confinement situation, was an odd and inexplicable situation, and how positive it has been to meet online to do the DLGs for their feelings of safety:

Many did not understand what was happening, why they were inside the house. And then, this moment of meeting everyone (because they did not understand why they were at home), seeing themselves on the screens and explaining to them that we are here… they have understood the situation, because it was being experienced in this way (TE1).

Reflections and dialogues around other sociological issues that occurred in these spaces are also important and protective in these confinement times, “this is a place for dialogue, for talking about violence, attraction toward no violence and preventive socialization, that is very important now” (PP1).

Teachers underline the importance of counting on spaces for dialogue and interaction, especially for students who cannot rely on peers at home in the form of siblings or other relatives of their age. “Maintaining a space for dialogue is very important for the diversity of students, because there are children who are alone, do not have siblings, and they don’t have the power to create dialogues, to interact” (TP4). All children need to share their concerns, their feelings, their routines with their friends and teachers, as is usually done in school, and according to families, the DLGs improve the communication with friends and teachers openly, and their relationships, as the school community is considered as a second family for many of them:

The fact that he meets with his classmates, since they cannot meet, makes them very happy. It is their second family and they need that (…) they benefit from seeing their mates. (…) They feel empowered and they are happy because they are being listened to. It is important to continue promoting this bond with their teachers, their classmates… I think the DLGs provide these spaces so that they can communicate more with colleagues (…) She [daughter] needs that moment of recognition, and not only for her, to see her classmates, to see how they are. and to share those moments (FE1).

For me, the DLGs [in this time of confinement] have provided me with a space of happiness. I have a great time in the DLGs, I think it gives me and my colleagues a lot of joy and happiness. Friendship with friends is very important, and [DLG] helps me in friendship with my friends and the teacher (SP4).

And this [undergo DLGs online] is very important because families alone cannot, cannot be teachers, because we do not know and because we cannot. We try but we cannot, it is impossible to get there (FP4).

Quality social interactions, crucial for well-being, take place in confinement through online DLGs. According to teachers, it is clear that the DLGs make possible the creation of quality relationships which have impacted on their students’ well-being now and for the future, and have opened options for new contexts of safe interactions:

We ensure the super quality [in DLGs sessions and interaction] and I think that in this sense the DLGs are benefitting the students a lot at this moment [confinement] for their development and their well-being (PP1).

The gatherings have been able to open other relationships and other opportunities to relate, that have created quality relationships and a safe context with an impact on their future (CS1).

### Feelings and the Emotional Dimension

It has been generally acknowledged by all the participants that DLGs have a remarkable impact on the emotional well-being of students, which has been increased in confinement times. DLGs are considered a space for interconnection, mainly in the emotional dimension, and in that place the deepest feelings are shared. A teacher expresses that he has set other spaces with his students through virtual calls that have not been as successful and meaningful as the DLGs. Moreover, in a moment of outbreak and isolation, the opportunity to share feelings and to be in touch with “the second family” have been valued as a key condition for child well-being:

The DLGs are an environment of interconnection but, above all, of emotional interconnection. It happens that all the stories in the end it happens that we address feelings, emotions and such. And oddly enough, although I have done other videocalls with the students so that they have other moments to talk as well and no [it doesn’t work]! In other moments there is no excuse. If it is not in DLG, after 5 min, no one knows what to talk about anymore, it’s like there is no reason to be there, together, there is no connection (.) I don’t know what it is, what I know for sure is that, indeed, with DLGs something happens that would not happen in other circumstances. That, in confinement, must be maintained (PP1).

To highlight all the emotional part of DLGs because children as my daughter, who have functional diversity, find difficulties to communicate, but above all to transmit and recognize feelings. So, what I would highlight the most is that these [online DLGs] are helping to understand and share feelings. It has been a great help for my daughter. (…) They have been all week without seeing each other, without seeing her other family (…) they are excited that they are going to see their classmates (FE1).

The DLGs nurture a space for sharing, apart from opinions, views, and knowledge, their emotions and feelings. Students have the opportunity to be in a context where to express freely and without judgment their positive, but also the negative emotions and concerns that maybe, in other spaces, they are not able to share. Thus, some teachers report that children often establish relationships between what is happening in the classical book and in their reality, telling their fears and worries through the characters of the book. But also, with teachers, some students have noticed that the line that separates the relationship of student-teacher with friendship is thinner now because during DLGs they have seen their teachers’ emotions, feelings and opinions as “peers”:

Now, with Oliver Twist in all the DLGs, the relationship with the current situation emerges. I think it is a space that helps them a lot to talk about those fears, or those concerns that perhaps they do not even dare to speak [out of DLGs] but empathizing with the characters of the book, yes, these issues appear and appear (FP3).

That moment, [the teacher] is like your friend, because you can tell him or her about your life… they are reading the same book as you and sharing ideas, their thoughts and so on… I like it because it’s like we already passed that little line between teacher-student (…) I also like to have volunteers, they can become your friends and you can speak as normal as if you had known them your entire live (SP1).

Dialogic Literary Gatherings have traditionally open methods to encourage the emergence of feelings and the expression of them, something which has been particularly relevant in this pandemic situation. A parent thanks online DLGs as they have helped his daughter to bring out and express the feelings she has kept inside “they bring out feelings that otherwise it would be difficult, and at that moment [during DLGs] they do it and tell you” (FP3). In this respect, a student recognizes how the book they have been reading has helped him to overcome the concern and to feel empathically through the links between the fiction story and the experienced situation:

I am comparing it [the book] with this situation of confinement, as in the book they [the characters] had to be so controlled, they couldn’t be mixed, no different thoughts were allowed… and that seemed to us quite similar to now. Now, we are supervised, we cannot go down into the street, we cannot touch each other, no hugs… and we started to compare and the truth is that you realize lots of things, and then you say ‘it just seems that I am inside the book! How did I get inside the book, in what moment?’ (…) Our teacher is who guides us and puts us on track and gives us examples for us to know, that it is not just fiction, that it is a reality and that these things can happen (SS1).

An increase of self-confidence has been highlighted by some teachers as one of the most noticeable changes in confinement times. The fact of being online may have provided them the opportunity to open up and to express their opinions without shyness. To provide some examples, a teacher talks about a girl with especial educational needs who did not used to participate in DLGs and who has started to be involved in and give her opinion much more frequently. The same observation is expressed by her mother, who points to the fact of doing online DLGs as the reason why children are gaining confidence to participating:

A student with special educational needs who hardly ever participated in the DLGs, since we have been in the online DLGs it happens that we are more in touch with the family, we know that she is fine (…) and the fact that she does not have a camera [in the electronic device where she does the online connection] and she can feel more confident, also she can have an adult there, [it is shown that] she participates much more in the DLGs (TP3).

I have realized that maybe as everyone is at home in front of their screen, they feel more confident and, children who did not used to participate so much in the face-to-face classes, they participate a lot now! (FP3).

Motivation has been another of the more commented aspects in the dialogues with participants, which has been very important to overcome confinement with a good state of mind in children. Students try to find the way to get connected to the DLG meetings and more and more students get involved in the sessions. A case was reported of a girl with an autistic disorder who found it difficult to be in all of the on-site DLG sessions, and now, in confinement this girl has started to be more motivated, sharing this moment with her classmates, connecting always to the DLG and staying there for the whole session:

[The case of] a student whose devices didn’t run the audios, also super-shy. So, this girl started to write in the chat because she, despite her shyness. she wanted to be there, because she wanted to be in the DLG. (…) Online DLGs started with 13 [students], then 16, then 23, that is, the number of people connecting has been increasing (TP4).

An autistic girl that I have in the classroom, in on-site DLGs never made it through the whole DLG, she had difficulties…, but now, her mother told me that she is super motivated (…) Now she connects to everything. and she is in the DLG all the time, she does her things, but she doesn’t leave the screen (TP4).

Friendship as one of the most important feelings is promoted in many ways through the DLG’s online meetings. Students’ stories, but also teachers’ and families’ interviews, have highlighted the condition of friendship as directly related to wellness and one of the first conditions they point out when they are asked about child-wellbeing, together with the emotional dimension:

Friendship [has enhanced] too, because now [with DLGs] we can see each other, and that means a lot to me. At the beginning [of confinement] we could not meet, neither see nor talk to each other, we could only see the photos we have [of friends] or to think about how they would be doing… but now we can see them in reality, what they are doing! (…) To see your friends in a DLG, and to be able to speak [with them] can make you feel better (SP1).

Self-esteem can be boosted after the participation in DLGs meetings, as students participate, give their point of view, and their statements enrich and can trigger new interventions:

After doing the DLG, she [daughter] feels very good, because she feels that her contribution has been appreciated by everyone and that sometimes it leads to more interventions. (…) She feels better and happier, her self-esteem grows (FP3).

The feeling of happiness has been shown through the stories and interviews. As a teacher states, families tell them that online DLGs is the happiest moment of the week: “And then the calls we have received from families in which they tell us that it [DLGs] are the happiest moment of the whole week for the kids. They are waiting for us to have the DLGs” (TS1). Families themselves express their children’s happiness in the interviews: “It [online DLGs] makes her happy, because she loves to interact with her peers!” (FP3).

### Impact at Home, and Homes That Make Impact Possible

One important factor has been identified in families that has enabled the improvements on the aforementioned dimensions. A mother expresses that when she herself gets involved in the online DLG, her daughter seems to have a better performance and seems happier: “I think that the days when I can participate in the DLG (…) she interacts more, and she feels happier” (FP4). Some teachers indicate some cases of children with educational needs or disorders that have improved a lot since confinement, due to having their relatives closer and helping them to formulate ideas or to feel confidence:

I have noticed improvements regarding their interventions thanks to the help and support of the families. One case is that of a boy who has a language disorder, who hardly ever participated in class, and only since DLGs started to participate, and now much more, in less time… and I think it is because his mother is close to him and helps him, gives him more confidence. And then the case of a child who also started with many reading difficulties that I have also noticed a lot of improvement now. (…) I associate it to being directly related to the help they get from their families, (…) they help them to link the ideas, or if they lose the thread, I hear how they get hooked on the idea again (TP2).

The fact of having more time for getting involved in their children’s activities may have been the reason why children are enhancing their development, improving learning and behavior, as this teacher explains. Her student, whose parents normally work all day and had no time to share with their son, now state that they, with the confinement situation, spend more quality time with their child and as a result, the behavior disorders and his academic performance have improved significatively during this time:

The situation has changed a lot because they [parents] have gone from not to being able to participate [in school life] and having to find other means of taking part, to now [with more time to spend with their son]. And I do totally link the fact that the child is much better thanks to the fact that his [parents] participate. (…) They say a lot that they are trying to give their child quality time and I think this is directly linked to the improvement of the boy… which in this case was a boy with a lot of behavioral problems and a very low level of academic performance… and now he has undergone quite a significant change! (TE1).

At home, an impact has been reported too. The new situation has allowed more time to be spent with the family, but the way this time is shared can have different consequences. In the case of DLGs, the online implementation has helped to have a positive impact on homes. For instance, the case of a very shy student who has been helped by her sister, a former student of the school very accustomed to on-site DLGs, that has encouraged her to participate more, and it was acknowledged by the whole class (students and teacher), congratulating her:

Alliances also arise, as in the case of a very shy student (…). Through the alliance she has with her sister, who had been a former student of mine and who is used to DLGs, (…) and through the communication with their the mother who said “well, let her be with her sister, it might encourage her, and that fact generated that she, my current student, participated, and that is cool! (…) the fact that she has been able to participate because her sister was there with her. Then we publicly congratulated her sister (CS1).

Families report how the preparation for and the participation in DLGs have not only strengthened parent-child bonds, but also allowed children to access more topics, deep conversations and ways of expressing feelings at home. Children also express how they like to share this time with their parents and learn from them: “I like that he [father] tells me… because sometimes I learn things from what he says, I ask him the meaning of a word and I also learn things from what he explains to me” (SP4). According to families, they enjoy working together with their children and take advantage of the debates that have emerged through the preparation of DLGs:

It’s funny about all the topics can emerge in a reading and that we can deal with at home, thus helping in their own learning and also in our family living. (…) At home, we try to work together in DLG, it is a time when we take the opportunity to talk a lot and express feelings (FP3).

From the first moment you have to sit down to read a story, starting with the bond you have to create as a mother-daughter to prepare it, because you know that there will be a session dedicated to that (…) Then you can integrate those explanations into your conversations with your children and that’s good because it helps you to talk more with them (FS1).

### Online DLGs for the Future. Overcoming Barriers for the Benefit of All

The challenge that some teachers and parents have reported is related to technology as a barrier. A teacher stated that there are students that feel less comfortable in front of a camera. One of the parents pointed to the fact that, in the first sessions, there was difficulty on respecting their turn to speak. One student said that she had the impression that the online version of DLG may be slightly slower. Nevertheless, all of them state that these challenges were present in the first sessions and that they have been getting used to the electronic devices and learning to cope with connection issues. In fact, according to some teachers, DLGs are online activities that work better and show results: “the proposal to have a DLG online in the confinement is the only thing that has worked for me, the only thing! Everything else, if I have explained, or made videos. I have no guarantees that it has worked, but the DLGs do.”

Students, families and teachers claim that online DLGs should be extended beyond confinement. Some of them, mostly students, propose to meet with other schools that implement DLGs through virtual means, making it possible to know new opinions, new views, and new possible friendships: “I think it would be cool and it could be done, because you can meet many people also from other schools (…), you can know what they think, their feelings.” (SP3, SP4, SS1). Other participants point out the possibility to extend DLGs in vacation times or beyond school hours. And some of them want its continuity in order to make it easier for families, even for volunteers, to participate, but also to make the most out of all the benefits that broader DLGs bring, as it has been demonstrated in this confinement time and reflected along this study:

For us [teachers], this confinement has been a gift, to be able to participate with all the families at the same time, having the opportunity to do DLGs with another school… I never imagined that we could do this from home, and with their families, that we could be interconnected. And now I think about it and I say why not? (…) It is so enriching, and I think that this does open up views of a good future. We love each other so much and we put up the barrier that we are far away… we have other tools, so for these tools that are the new technologies, we can “go” [virtually] to our school and forget about the pain of the distance, we can have virtual volunteers, virtual families… and to take the DLGs outside the school hours, and we will do them [DLGs] at a time that everyone can… and we will meet again (TP4).

## Discussion

This study has analyzed an evidence-based action, the DLGs, replicable in any educational context, which already had evidence of its effectivity in the face-to-face format. It has been recommended into European public policy and other contexts and now it is transferred to home spaces through online connection. The results of the research have shown how the implementation of DLGs in online format, transferring them to homes, is having an impact on the improvement of children’s well-being, from a subjective view. Precisely, the improvements have taken place in some of the well-being dimensions, such as the emotional, educational and social ones, reducing the risk of anxiety regarding the new situation of confinement and all the inputs children receive related to the COVID-19 outbreak. These virtual spaces have shown how they enable the involvement of families for which participating had never been possible before and have allowed many children to participate more and more thanks to the facilities provided by virtual communication. The study has gone further, exploring and revealing the benefits of this action for family life and in their homes, and how the reciprocal collaboration of the community agents has enabled such impact. It is concluded that online DLGs enable an effective management in the protection of children’s well-being, which is also accessible, in terms of resources and natural environment.

The literature review has shown how the understanding of child well-being as a concept implies a multidimensional view (Statham and Chase, 2010) and that it cannot be separated from the influence of the community interactions with teachers, family, friends and classmates. In the same line, results of this study shed light on a way to maintain meaningful interactions, through an evidence-based action transferred to homes, which has an impact on different levels—particularly social, emotional and educational—keeping and even improving a feeling of well-being in such an extreme situation as this COVID-19 pandemic.

To obtain these results and following the international recommendations, the study has been approached from a communicative perspective of research (Puigvert et al., 2012). Subsequently, the focus of the inquiry has been the subjective dimension of well-being, accounting for the participants’ views and thoughts, and adapting the research techniques to facilitate the most beneficial for both, researcher and participant. Subjective well-being, associated with a sense of happiness or life satisfaction (Dinisman and Ben-Arieh, 2016) has been widely reported by all the interviewees, as shown in the “Results” section, and the quality of interpersonal relationships (Diener et al., 2018) that take place in preparing for and participating in online DLGs is highlighted as the main factor facilitating said satisfaction.

The results of this study are supported by recommendations such as those from the Centre on the Developing Child and Harvard University (2020), where the idea of constructing community for improving well-being is emphasized. Protecting against the toxic stress through virtual contact with friends and supporting families during the outbreak and further are ideas stressed in the recommendations and reinforced by our study. Because the ultimate goal is to promote long-term well-being, both for children and their families, society and communities need to support responsive care in different settings, either school or home, but together, in community (Center on the Developing Child, and Harvard University, 2014).

Considering the feasibility of the implementation of online DLGs, and the fact that the impact of traditional DLGs has been collected in policies and recommendations, it is relevant to assess the opportunity to set it as a possible public policy in educational institutions during the time of the COVID-19 pandemic, in order to facilitate a wider impact on well-being for more children. The development of policies addressed to ensure a healthy child development has been studied as the foundation of a productive society intended to create a successful future. Public programs and policies directly affect the community capacities for strengthening that healthy development, underpinned by safe contexts and quality stable relationships (Center on the Developing Child, and Harvard University, 2014). According to Wang et al. (2020), it is the responsibility of not only families or schools, but also of governments, to immediately act to avoid, as far as possible, the impact of the COVID-19 pandemic on children’s physical and mental health (Wang et al., 2020). Taking this into consideration and knowing the potentialities and viability of DLGs in homes, it seems possible and convenient to contemplate the proposal of developing public policies in this direction. Ensuring technological availability and internet access for conducting the DLGs needs to be considered in order to transfer this educational action.

This study has been limited to the analysis of subjective well-being, given the urgency of the crisis and the need to adjust the research to the available sample. The online DLGs were introduced less than a month before the study, not before ensuring technical support for every family without internet access or technological resources in order to ensure equal terms for all to share the educational experience. This time of deployment makes it difficult to measure the impact of DLGs transferred to the home on aspects related to the child’s objective well-being. This study has shown that an impact on child well-being occurs and, therefore, further research on the elements and strategies of DLGs that facilitate and promote this impact is needed in order to facilitate the transference of this action to other educational spaces. Considering their further implementation beyond the confinement situation, it would also be relevant to delve into the long term impact of implementing this action, especially to explore its possible effects on improving academic performance, or preventing school failure. Further research on how the implementation of other Open Doors Actions (Roca et al., 2020), such as Dialogic workspaces with students, teachers and volunteers; Class assemblies, or other Dialogic Gatherings (musical, artistic, scientific, etc.), could have an impact on child well-being during the time of pandemic crisis and school closing would be relevant. A future line of research on the benefits of extending online DLGs after the confinement situation, in the upcoming contexts of the new normal in the education domain is also of interest.

The uncertainty which permeates the new global situation provides little insight about whether social distancing, teleworking and home schooling are new realities that will continue to become more and more common in our societies. What seems probable is that it will go along to become the new normal. Results of the study indicate the desire—once the online connection obstacles are overcome, and the potentialities discovered—for continuing to implement DLGs online in the future. Considering the new setting and the voices of participants, who expressed their desire to continue with this action regardless of the pandemic situation, and given its complementarity with the face-to-face format, it seems plausible to think that this educational action will live on beyond the outbreak, as a useful and successful tool for boosting children’s education and, above all, children’s well-being as well as their families’, encouraging the creation of a wider community and broader participation. Once the data collection period for this study was finished, researchers learnt that more and more between-schools alternatives had been emerging once the potentialities and opportunities offered by a virtual format of DLG were discovered. The different schools found in this online action an opportunity to connect different learning communities virtually, broadening educational horizons and extending their impact, through the joint implementation of this new Open Doors Action.

## Data Availability Statement

The raw data supporting the conclusions of this article will be made available by the authors, without undue reservation.

## Ethics Statement

The studies involving human participants were reviewed and approved by Ethics Board of the Community of Researchers in Excellence for All (CREA), University of Barcelona. Written informed consent to participate in this study was provided by the participants’ legal guardian/next of kin.

## Author Contributions

LR-E and ER-C conceived the original idea. SL-J conducted the literature review. ER-C coordinated the data collection and conducted the interviews. SL-J transcribed and analyzed the results with the support of ER-C and MR-S. SL-J wrote a draft of the manuscript. ER-C and MR-S revised it and included corrections. LR-E revised the final version of the manuscript. All authors contributed to the article and approved the submitted version.

## Conflict of Interest

The authors declare that the research was conducted in the absence of any commercial or financial relationships that could be construed as a potential conflict of interest.

## Footnotes

1 The Ethics Board was composed by: Dr. Marta Soler (president), who has expertise in the evaluation of projects from the European Framework Programme of Research of the European Union, and of European projects in the area of ethics; Dr. Teresa Sordé, with expertise in the evaluation of projects from the European Framework Programme of Research and researcher in the area of Roma studies; Dr. Patricia Melgar, founding member of the Catalan Platform against gender violence, and researcher in the area of gender and gender violence; Dr. Sandra Racionero, former secretary and member of the Ethics Board at Loyola University Andalusia (2016–2018), and review panel member for COST action proposals in the area of health; Dr. Cristina Pulido, expert in data protection policies and child protection in research and communication and researcher in communication studies; Dr. Oriol Rios, founding member of the “Men in Dialogue” association, researcher in the area of masculinities, as well as editor of “Masculinities and Social Change,” a journal indexed in WoS and Scopus; and Dr. Esther Oliver, who has expertise in the evaluation of projects from the European Framework Programme of Research and is researcher in the area of gender violence.
